# Treatment outcomes for isoniazid-monoresistant tuberculosis in Peru, 2012-2014

**DOI:** 10.1371/journal.pone.0206658

**Published:** 2018-12-04

**Authors:** Jose Gabriel Cornejo Garcia, Valentina Antonieta Alarcón Guizado, Alberto Mendoza Ticona, Edith Alarcon, Einar Heldal, David A. J. Moore

**Affiliations:** 1 Hospital Nacional Arzobispo Loayza, Lima, Perú; 2 Dirección de Prevención y Control de Tuberculosis, Ministry of Health, Lima, Peru; 3 Hospital de Emergencias Villa El Salvador, Ministry of Health, Lima, Peru; 4 Pan American Health Organization, Washington, D.C., United States of America; 5 The International Union Against TB and Lung Disease, Oslo, Norway; 6 TB Centre, London School of Hygiene and Tropical Medicine, London, United Kingdom; 7 Universidad Peruana Cayetano Heredia, Lima, Peru; University of Cape Town, SOUTH AFRICA

## Abstract

**Background:**

Resistance to isoniazid is the most common form of drug-resistance in tuberculosis. However only a tiny proportion of TB patients in the world have access to isoniazid drug susceptibility testing—the widely implemented Xpert MTB/RIF technology only tests for resistance to rifampicin. Patients with isoniazid mono resistance that is not identified at baseline are treated with a standard regimen that effectively results in rifampicin mono-therapy during the latter four months of the six month treatment course, exposing remaining viable organisms to a single agent and greatly increasing the risk of development of multi drug-resistant TB. Unusually, Peru has pioneered universal pre-treatment drug susceptibility testing with methods that identify isoniazid resistance and has thus identified a large number of individuals requiring tailored therapy. Since 2010, treatment in Peru for isoniazid-resistant tuberculosis without multidrug-resistant tuberculosis (Hr-TB) has been with a standardized nine-month regimen of levofloxacin, rifampicin, ethambutol and pyrazinamide. The objectives of this study were to evaluate the outcomes of treatment for patients with Hr-TB initiating treatment with this regimen between January 2012 and December 2014 and to determine factors affecting these outcomes.

**Methods:**

Retrospective cross-sectional study; case data were obtained from the national registry of drug-resistant tuberculosis. Patients diagnosed with isoniazid resistant TB without resistance to rifampicin, pyrazinamide, ethambutol and quinolones as determined by either a rapid drug susceptibility testing (DST) (nitrate reductase test, MODS, Genotype MTBDRplus) or by the proportion method were included.

**Findings:**

A total of 947 cases were evaluated (a further 403 without treatment end date were excluded), with treatment success in 77.2% (731 cases), loss to follow-up in 19.7% (186 cases), treatment failure in 1.2% (12 cases), and death in 1.9% (18 cases). Unfavorable outcomes were associated in multivariate analysis with male gender (OR 0.50, 95% CI 0.34–0.72, p<0.05), lack of rapid DST (OR 0.67, 95% CI 0.50–0.91, p = 0.01), additional use of an injectable second-line anti-tuberculous drug (OR 0.46, 95% CI 0.31–0.70, p<0.05), and treatment initiation in 2014 (OR 0.77, 95% CI 0.62–0.94, p = 0.01).

**Interpretation:**

The treatment regimen implemented in Peru for isoniazid resistant TB is effective for TB cure and is not improved by addition of an injectable second-line agent. Access to rapid DST and treatment adherence need to be strengthened to increase favorable results.

## Introduction

Tuberculosis (TB) remains a significant health issue worldwide. Resistance to anti-TB drugs makes treatment difficult. Isoniazid (H) resistance without concomitant rifampicin resistance is a common problem. Inadequate management of isoniazid-resistant TB (Hr-TB) creates the ideal circumstances for amplification of resistance to multidrug resistance (MDR, resistance to at least isoniazid and rifampicin) which is much harder to treat. Detection of isoniazid resistance is often delayed or overlooked entirely, and even once identified there is very limited evidence to direct treatment regimen choice in such cases.

Worldwide, Hr-TB without MDR-TB is estimated at 9.5% of all cases; for new cases it is 8.1% and for previously treated cases it is 14% [1]. According to the National Surveillance Study in Peru (2005–2006), Hr-TB without MDR-TB rate in new cases is 6.3% and with previous treatment it is 6.7% [2].

Timely detection of this and other types of resistance is important; the World Health Organization (WHO) approved the End-TB Strategy in May 2014 one of the mainstays of which is the universal use of rapid drug susceptibility testing (DST) for early resistance detection [3]. In Peru these measures were adopted earlier; in 2002 drug susceptibility testing for first-line and second-line anti-TB drugs using the proportion method on Middlebrook 7H10 agar (APP) was implemented; by 2006 implementation and decentralization of nitrate reductase assay was initiated [4]; by 2008 MODS was validated [5]; and by 2010 the GenoType MTBDRplus method was validated by the National Institute of Health (Instituto Nacional de Salud, INS) [6]. This development of rapid drug susceptibility testing (DST) allowed for universal access since 2011, which in turn facilitated early resistance detection not only for rifampicin but also for isoniazid [7].

There is no international consensus for the treatment of Hr-TB, due to a lack of evidence; remarkably, however, most of the emphasis is on regimens that only include first-line drugs. The British Thoracic Society (BTS) recommended in 1998 2 months of streptomycin (S), rifampicin (R), pyrazinamide (Z), and ethambutol (E) followed by 7 months of rifampicin and ethambutol (2SRZE/7RE) when H resistance is diagnosed before treatment initiation, and 2RZE/10RE if the patient had already started treatment [8], a position maintained in the recommendations from the 2016 NICE clinical guidelines [9]. In 2003 the ATS (American Thoracic Society), IDSA (Infectious Diseases Society of America), and CDC (Center of Disease Control and Prevention), recommended HREZ and then RZE after H-resistance identification, for a total of 6 months [10].

In the 2008 Management Guidelines, the World Health Organization (WHO) recommended R,Z,E for 6–9 months, with addition of levofloxacin (Lfx) for extensive disease [11]; however, the 2010 Guidelines state that the most effective regimen for this type of resistance is not known, and recommend that for cases which had already started treatment and have known or suspected H resistance, HRE be maintained for 5–7 months, whilst acknowledging that the level of evidence supporting this or any other recommendation is insufficient [12]. In the 2014 Management Guidelines, the WHO again recommended R,Z,E for 6–9 months with caution, both if resistance to E and Z is not known, due to risk of multidrug resistance, and in case of resistance to H alone, due to potential for R resistance amplification; based on expert opinions, Lfx may be added as well [13]. In the latest 2016 Management Guidelines, the WHO makes no recommendations about Hr-TB management, due to lack of evidence [14]; new specific recommendations are expected in 2018.

In Peru, the 2006 Technical Standard for Tuberculosis Control recommended 2 months of SHREZ for H resistance alone, followed by 1 month of HREZ, and then 5 months of HRE twice a week (2SHRZE/1HRZE/5H_2_R_2_E_2_), and in case of H and S resistance, kanamycin (Km) was to be added to the regimen 2KmRZE/1Km_3_RZE/6RZE [15]. The 2010 Peru Technical Standard indicated RZE or RZE plus ciprofloxacin (Cpx) for 9 months, depending on the timing of diagnosis [16]; that same year, based on the 2008 WHO Management Guidelines, Cpx was replaced by Lfx in all treatment regimens [11]. The treatment regimen in the 2013 Technical Standard was two months of Lfx, R, E, Z for the intensive phase, followed by seven months of Lfx, R, E (2LfxRZE/7LfxRE)[17], which was progressively implemented. This regimen was designed based on review and analysis of susceptibility results from 12,311 *M*. *tuberculosis* isolates obtained from 2007 to 2009, in which 98.9% of H resistant non-MDR strains diagnosed by rapid DST were found to be susceptible to at least 3 agents of a CpxRZE regimen [18]. Prior to this 2013 recommendation, in some cases an aminoglycoside was added to the regimen, at the discretion of the treating clinician but with no clear justification recorded.

Unlike treatment for susceptible and MDR TB, there are few studies and no clinical trials which assess different regimens for Hr-TB without MDR-TB, and the recent review by Gegia [19] shows that first-line treatment only is inadequate; here we report treatment outcomes for a cohort of patients with Hr-TB treated under programmatic conditions in Peru with a standardized nine month quinolone containing regimen.

## Methods

### Study design and location

This is an operational, retrospective and descriptive study of a cohort of patients who received treatment for H-resistant TB without MDR-TB in Peru from January 2012 to December 2014.

In Peru, as stated in the 2013 Technical Standard [16], all diagnosed TB cases require a sample for rapid DST and culture. If the rapid DST shows isoniazid and/or rifampicin resistance, the APP DST is performed at the national mycobacteria reference laboratory at the INS for H, R, E, S, Km, PAS, Lfx, capreomycin (Cm), ethionamide (Eto), and cycloserine (Cs); in November 2014, Cpx susceptibility testing was replaced by Lfx. Additionally, susceptibility to Z is assessed using Wayne or MGIT assays; the national mycobacteria reference laboratory participates in the external quality assurance programme overseen by the supranational laboratory.

All cases with identified drug resistance are evaluated by Regional Retreatment Assessment Committees to approve treatment initiation. Those cases for which treatment was started based upon rapid DST results are subsequently reassessed using APP in order to ratify or change the regimen based on the complete susceptibility profile.

### Study subjects

All approved cases are reported and entered with a code to the National Resistant Tuberculosis Registry (Registro Nacional de Tuberculosis Resistente, RNTR); healthcare facilities where patients will receive treatment are required to report treatment initiation and termination, and must perform monthly microbiological surveillance control with sputum smear microscopy and culture. Bacteriology and DST are registered in the RNTR. Cultures and DST are also registered in NETLAB, the INS Laboratory Information System.

The cohort for evaluation included cases that fulfilled the following criteria:

- H resistance as determined by rapid DST (nitrate reductase, MODS, or Genotype MTBDRplus) or by the APP DST. If APP DST was available, H resistance should be confirmed, with no Cpx or Lfx, R, Z, and E resistance identified
- Treatment initiation and termination information available (although, for the purposes of assessing the representativeness of the evaluated cohort, data from cases with no treatment completion dates were also collected to compare “not evaluated” cases with the evaluation cohort)
- received Lfx, R, Z, E with or without an injectable drug (S, Km, or Cm)

The following cases registered in the RNTR were excluded:

- the APP DST demonstrated susceptibility to isoniazid
- the APP DST demonstrated MDR/XDR-TB
- patient initiated treatment for MDR-TB
- patient initiated treatment with second-line drugs due to adverse reactions to first-line drugs, a comorbidity or medical judgement without having isoniazid resistance

### Variables

The information for this study comes from the RNTR. The code for each case was entered and the following variables were obtained: gender, age, treatment initiation and termination dates, location of treatment administration, HIV and diabetes status, injectable drug use, rapid DST, APP DST and available culture results. For cases lacking complete information on cultures and susceptibility testing in RNTR, additional data was obtained from NetLab.

Each case was assigned a treatment outcome, but there are no established definitions for treatment outcomes using this regimen; therefore, for study purposes the 2013 WHO definitions were adapted [20]:

- Cured: requires two negative cultures, one during treatment and the other at the end of treatment.
- Treatment completed: treatment is finished with no evidence of failure but does not fulfill microbiological criteria for “cured”.
- Loss to follow-up: treatment was discontinued for over 30 consecutive days.
- Failure: positive culture at month 5 or later, or treatment change with at least two new drugs due to lack of conversion at the end of intensive treatment phase.
- Death: patient death for any cause during treatment.
- Treatment success: cured plus treatment completed.
- Not evaluated: a treatment outcome cannot be assigned as treatment conclusion has not been reported to the RNTR.

### Data collection and statistical analysis

The information collected was entered into an Excel Microsoft Office 2016 database. Cases were divided between cases with treatment outcome (evaluated) and those not evaluated; variables were compared in cases which received LfxREZ and LfxREZ plus an injectable drug within the evaluated and not evaluated groups, and then comparisons were made between the two groups for significant differences using Chi-squared test (X^2^). Then cases were divided based on treatment outcome. A univariate and multivariate logistic regression analysis was performed to determine whether any variable affected favorable treatment outcome. A p-value <0.05 was considered significant. The data was analyzed using STATA14 software (Stata corp, Texas USA).

### Ethical considerations

The study protocol was reviewed and approved by The Union Ethics Committee (International Union Against Tuberculosis and Lung Disease) and the Hospital Nacional Arzobispo Loayza (Lima, Peru) Institutional Research Ethics Committee.

## Results

**Fig 1:**
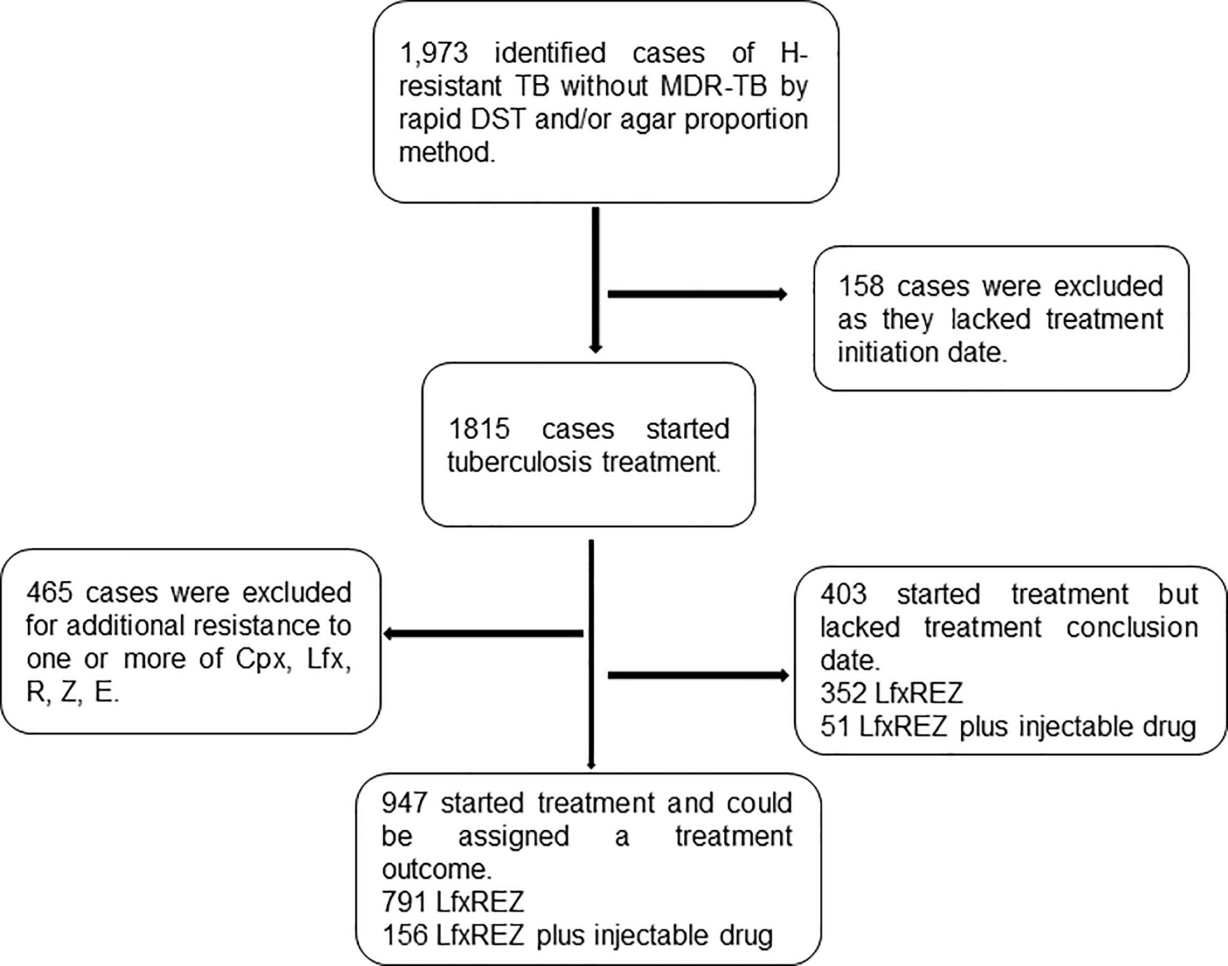
Flowchart for case inclusion.

In the RNTR, 1973 cases of H-resistant TB without MDR-TB were identified. A total of 623 cases were excluded; 1350 started treatment with LfxREZ (n = 1143) or LfxREZ plus an injectable drug (n = 207); of these, 403 cases had no treatment conclusion date, so treatment outcome could not be evaluated; these cases were designated as not evaluated. There were 947 cases with treatment initiation and termination dates (791 and 156 with and without use of injectable drug, respectively) where treatment outcome was evaluated (evaluated cases) (Fig 1).

### Evaluation cohort vs. not evaluated cases

To determine whether the evaluation cohort is representative of patients treated for Hr-TB, the characteristics of evaluated and not evaluated groups were compared. Table 1 shows these results; there were no significant differences in gender, comorbidities (HIV, diabetes), or availability of results from rapid DST and APP DST. Patients without a treatment outcome date, and thus not evaluated, were more likely to have been treated outside of Lima-Callao (p = 0.02) and have started treatment in 2013/14 (p<0.05). The 15–34 age group was also slightly over-represented amongst the non-evaluated (p = 0.03).

**Table 1:** Characteristics of evaluated and not evaluated groups.

		Evaluated cases n = 947		Not evaluated cases n = 403		p
		n	%	n	%	
Gender	F	295	31.1%	126	31.3%	1.0
	M	652	68.9%	277	68.7%	
Age	0–14	22	2.3%	8	2.0%	0.03
	15–34	603	63.7%	291	72.2%	
	35–54	210	22.2%	67	16.6%	
	>55	112	11.8%	37	9.2%	
Year	2012	316	33.4%	80	19.8%	<0.05
	2013	283	29.9%	143	35.5%	
	2014	348	36.7%	180	44.7%	
HIV	positive	41	4.3%	11	2.7%	0.3
	negative	806	85.1%	344	85.4%	
	Not evaluated	100	10.6%	48	11.9%	
Diabetes	Yes	69	7.3%	33	8.2%	0.6
	No	878	92.7%	370	91.8%	
Injectable TB drug	Yes	156	16.5%	51	12.7%	0.08
	No	791	83.5%	352	87.3%	
Location	Lima-Callao	614	64.8%	233	57.8%	0.02
	Provinces	333	35.2%	170	42.2%	
Rapid DST	H resistance	760	80.2%	331	82.1%	0.2
	Not available	159	16.8%	60	14.9%	
	Susceptible	27	2.8%	9	2.2%	
	MDR-TB	1	0.2%	3	0.8%	
APP DST	H-resistant	285	30.1%	120	29.8%	0.4
	HS-resistant	316	33.7%	114	28.3%	
	HSEto-resistant	109	11.5%	54	13.4%	
	HEto-resistant	64	6.8%	29	7.2%	
	Other	24	2.5%	8	2.0%	
	Not available	149	15.4%	76	19.3%	

### Characteristics of evaluated cohort (Table 1)

In the evaluated group, the most common resistance pattern was HS (33.3%), followed by H resistance (30.1%), HSEto resistance (11.5%) and HEto resistance (6.7%); this profile did not differ significantly from that of the group without outcome evaluation. Additional use of a second-line injectable anti-TB drug was significantly more frequent in in 2012 and 2013 (31% and 37% of cases) than in 2014 (1.5%), and usage was more common in Lima-Callao than in the provinces (p<0.05 for both, S1 Table).

### Treatment outcomes

Table 2 shows treatment outcomes and distribution based on study variables. Treatment success, a composite of cure and treatment completion, was recorded for 77.2% of cases. Although the percentages of patients experiencing failure (1.3%) and death (1.9%) were very low, the loss to follow-up was high (19.6%), with no significant variation over the years. Therefore, the unsuccessful treatment outcome in 22.8% was driven by loss to follow-up.

**Table 2:** Characteristics of evaluated cases (n = 947) based on treatment outcomes.

	Cured	Completed treatment	Loss to follow-up	Failure	Death	Total
All	326 (34.4%)	405 (42.8%)	186 (19.6%)	12 (1.3%)	18 (1.9%)	947 100%
Sex						
F	108 36.6%	143 48.5%	38 12.9%	1 0.3%	5 1.7%	295 (31.1%)
M	218 33.4%	262 40.2%	148 22.7%	11 1.7%	13 2%	652 (68.9%)
Age (years)						
0–14	7 31.8%	12 54.5%	2 9.1%	0	1 4.6%	22 (2.3%)
15–34	210 34.8%	248 41.1%	130 21.6%	8 1.3%	7 1.2%	603 (63.7%)
35–54	70 33.3%	93 44.3%	37 17.7%	3 1.4%	7 3.3%	210 (22.2%)
>55	39 34.8%	52 46.4%	17 15.2%	1 0.9%	3 2.7%	112 (11.8%)
Year						
2012	91 28.8%	162 51.3%	55 17.4%	1 0.3%	7 2.2%	316 (33.7%)
2013	109 38.5%	104 36.7%	60 21.2%	1 0.4	9 3.2%	283 (29.9%)
2014	126 36.2%	139 39.9%	71 20.4%	10 2.9%	2 0.6	348 (36.4%)
HIV						
Positive	11 26.8%	10 24.5%	16 39.0%	1 2.4%	3 7.3%	41 (4.3%)
Negative	286 35.5%	351 43.5%	148 18.4%	9 1.1%	12 1.5%	806 (85.1%)
Not evaluated	29 29.0%	44 44.0%	22 22.0%	2 2.0%	3 3.0%	100 (10.6%)
Diabetes						
Yes	18 26.1%	34 49.3%	13 18.9%	3 4.3%	1 1.4%	69 (7.3%)
No	308 35.1%	371 42.2%	173 19.7%	9 1.1%	17 1.9%	878 (92.7%)
Location						
Lima	243 39.6%	219 35.7%	132 21.5%	7 1.1%	13 2.1%	614 (64.8%)
Provinces	83 24.9%	186 55.9%	54 16.2%	5 1.5%	5 1.5%	333 (35.2%)
Rapid DST						
H resistance	277 36.4%	321 42.2%	142 18.7%	8 1.1%	12 1.6%	760 (80.2%)
No rapid DST Available	42 26.4%	71 44.7%	38 23.9%	3 1.9%	5 3.1%	159 (16.8%)
Susceptible	6 22.2%	13 48.2%	6 22.2%	1 3.7%	1 3.7%	27 (2.9%)
MDR-TB	1					1 (0.1%)
APP DST						
H resistance	84 29.5%	125 43.9%	67 23.5%	3 1.0%	6 2.1%	285 (30.1%)
HS-resistant	100 31.6%	150 47.5%	58 18.3%	4 1.3%	4 1.3%	316 (33.4%)
HSEto-resistant	54 49.6%	36 33.0%	14 12.8%	2 1.8%	3 2.8%	109 (11.5%)
HEto-resistant	26 40.6%	18 28.1%	19 29.7%	1 1.6%	0	64 (6.8%)
Other	5 20.8%	17 70.8	1 4.2%	0	1 4.2%	24 (2.5%)
Not available	57 38.3%	59 39.6%	27 18.1%	2 1.3%	4 2.7%	149 (15.7%)
Injectable						
Yes	54 34.6%	51 32.7%	40 25.6%	0	11 7.1%	156 (16.5%)
No	272 34.4%	354 44.8%	146 18.5%	12 1.5%	7 0.8%	791 (83.5%)

Women experienced greater treatment success than men (85.1% vs. 73.6%), with a large gender differential in dropout and failure rates for men vs. women (22.7% vs. 12.9% and 1.7% vs. 0.3%, respectively), with similar death rates (2.0% in men and 1.7% in women). Loss to follow-up was twice as frequent amongst patients with HIV co-infection (39.0 vs. 18.4% amongst HIV-uninfected).

**Fig 2:**
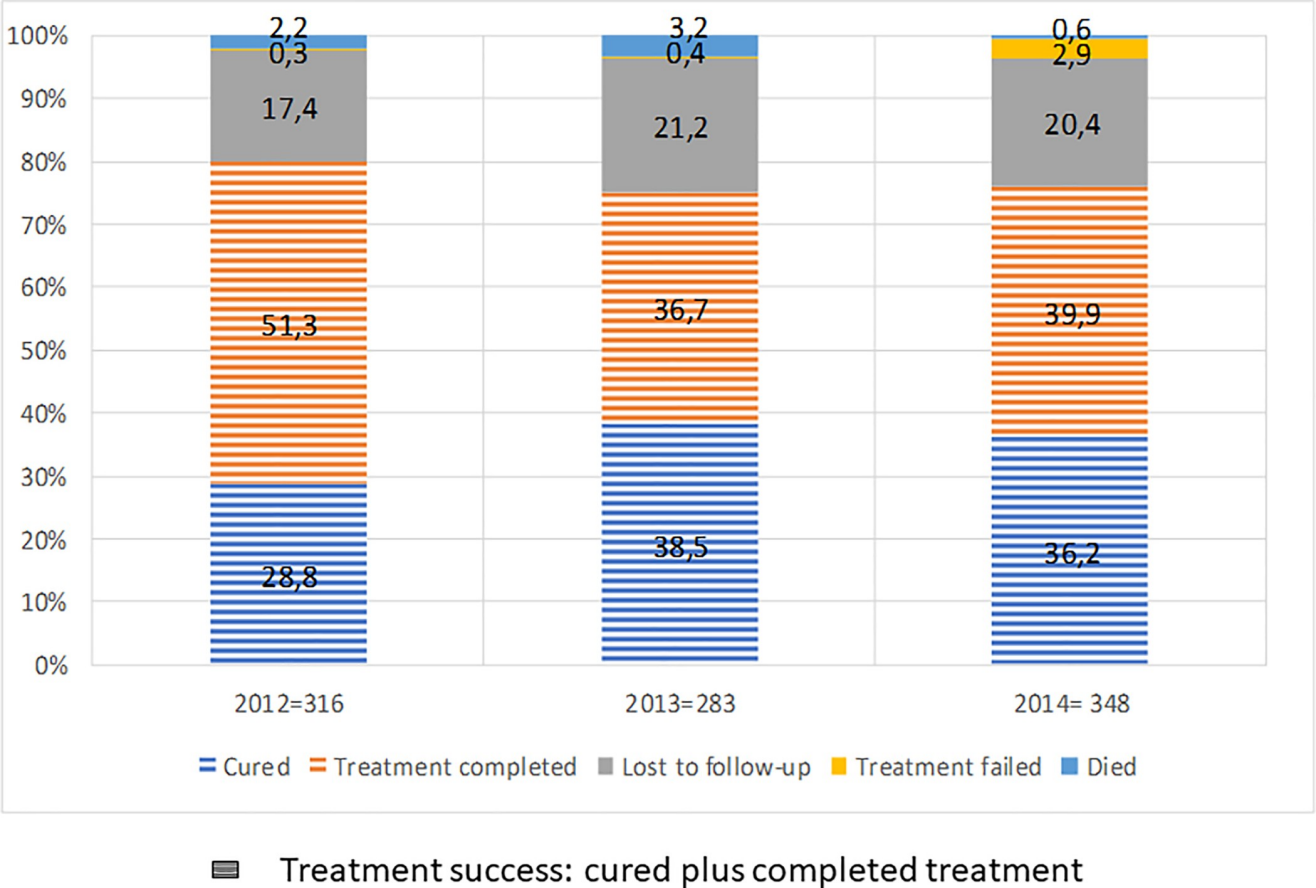
Treatment outcome based on treatment initiation year.

Amongst patients for whom H resistance was only diagnosed with the conventional agar proportion method–those without an available rapid H DST–the percentage of loss to follow-up (23.9% vs. 18.7%), failure (1.9% vs. 1.1%) and death (3.1% vs. 1.6%) was higher than for patients with available rapid DST results. Use of a second-line injectable (vs. non-use) was associated with a higher percentage of loss to follow-up (25.6% vs. 18.5%) and death (7.1% vs. 0.8); among treatment failure cases there were no patients who received second-line injectable treatment. Fig 2 shows treatment outcomes by year of treatment initiation. Treatment success was recorded for 80.1% in 2012, 75.2% in 2013, and 76.1% in 2014.

The univariate logistic regression analysis (Table 3) showed decreased treatment success to be associated with male gender, lack of an available rapid DST for H resistance and use of a second-line injectable drug. In the multivariate logistic regression analysis, in addition to the previous variables, treatment initiation in 2014 was also independently associated with a less favorable treatment outcome.

**Table 3:** Univariate and multivariate logistic regression analysis of variables that may affect treatment success.

	Univariate analysis OR (95% CI)	p	Multivariate analysis OR (95% CI)	p
**Sex**				
Female	Reference			
Male	0.48 (0.33–070)	< 0.05	0.50 (0.34–0.72)	< 0.05
**Age (years)**				
0–14	Reference			
15–34				
35–54				
>55	1.00 (0.99–1.01)	0.7		
**Year**				
2012	Reference			
2013				
2014	0.89 (0.74–1.07)	0.2	0.77 (0.62–0.94)	0.01
**HIV**				
Yes	Reference			
No				
Not evaluated	0.97 (0.92–1.03)	0.4		
**Diabetes**				
Yes	Reference			
No	1.01 (0.95–1.07)	0.7		
**Treatment location**				
Lima-Callao	Reference			
Provinces	0.72 (0.52–1.01)	0.05		
**Rapid DST**				
H-resistant	Reference			
Not available				
Susceptible				
MDR-TB	0.74 (0.55–0.99)	0.05	0.67 (0.50–0.91)	0.01
**APP DST**				
H-resistant	Reference			
HS-resistant				
HSEto-resistant				
HEto-resistant				
Another resistance				
No APP available	1.01 (0.96–1.06)	0.5		
**Injectable use**				
No				
Yes	0.54 (0.37–0.79)	0.001	0.46 (0.31–0.70)	< 0.05

Variables with p≤0.2 on univariate analysis were included in the multivariate model; for clarity, only those variables found to be statistically significantly associated with treatment outcome are shown in the table.

## Discussion

This study has demonstrated that the LfxREZ regimen indicated for Hr-TB in Peru results in a favorable outcome in 77.2% of treated patients, with the dominant reason for lack of treatment success due to a high proportion of loss to follow-up. Loss to follow-up has been a programme-wide problem in Perù, not specific to Hr-TB; between 2008–2010 the loss to follow up was 10% for drug-susceptible TB and 18–20% for MDR TB [7]. Variables that diminished treatment success were male gender, lack of available rapid DST for H resistance, use of adjunctive second-line injectable drug therapy and treatment initiation in 2014.

A strength of this operational research study is the usage of nationwide programmatic data from the National Resistant Tuberculosis Registry and inclusion of all cases approved for treatment initiation for isoniazid-resistant TB. In addition, the strict microbiological inclusion criterion depended upon proven isoniazid resistance at the national mycobacteria reference laboratory, which is overseen for EQA by the supranational reference laboratory.

The registry includes a significant section (403/1350, 30%) of patients for whom no treatment outcomes (“not evaluated”) had been recorded. To assure that exclusion of these patients from the evaluation cohort would not generate a major bias, their characteristics were analyzed and found to show no important differences regarding demographic data and disease characteristics. It has only been shown that compared with evaluated patients, “not evaluated” status was more common in the provinces than in Lima and were more frequent in 2013 and 2014 than in 2012. In the evaluation cohort, loss to follow-up was more common in the two more recent years of study. Despite the apparent demographic and clinical similarity of the “not evaluated” group to the “evaluated” group, there remains a significant concern that the evaluated group may not accurately represent the entire cohort of LfxREZ treated patients. From our available data we are unable to exclude the possibility that deaths, treatment failures and loss to follow-up may have been much greater in the “not evaluated” group, which remains an important limitation of this analysis.

Treatment success broadly consisted of half cured and half patients who had completed treatment. Although failures and deaths were very few, the high proportion of loss to follow-up is striking, and it is important to acknowledge the possibility that there may have been more patients who failed treatment or died amongst this group that were not captured or recognized by the TB programme.

Men had less favourable outcomes and more loss to follow up than women, as is seen for TB in general, regardless of drug-susceptibility. In Peru, TB mainly affects men and the age group 15–44, and 95% of cases are seen in the lower socioeconomic strata, where men are the ones who mainly work; this may in turn affect treatment adherence and lead to subsequent unfavorable outcomes, since work or school are important reasons for non-adherence [21].

Lack of an available rapid DST for H resistance, and thus reliance only on the APP DST result with longer turnaround times, may delay initiation of an adequate treatment and thus explain lower favorable outcomes. Current implementation in Peru of universal rapid DST, which includes isoniazid susceptibility testing–MODS and Genotype MTBDRplus–has been a key policy for treatment success, with a standardized regimen initiated without delay (Obregon Boltan G. et al. Introduction of rapid drug susceptibility tests and treatment outcomes for multidrug-resistant TB in Peru, 2010–2015. In Press Int J Tuberc Lung Dis 2018). A drawback to the Xpert MTB/RIF test, implemented at large-scale in several countries, although not yet in Peru, is the lack of information about isoniazid resistance. Without these rapid DSTs, patients included in this analysis could not have been identified; most patients would have started 2RHZE4RH, which in a recent systematic review [19] was associated with failure in 11% and relapse in 10% of cases, and with additional acquired resistance to rifampicin (acquired MDR-TB) in 8% of cases. It may be inferred that this source of MDR-TB has been shut off with the implementation of rapid DST in Peru.

In 16% of cases, a second-line injectable drug was added to the standardized regimen (LfxREZ). Among the 18 deceased patients, 61% received an injectable drug, which suggests that the use may have been indicated for cases with a greater clinical deterioration (as this was not algorithm-based but based on medical decision).

Treatment initiation year was identified as a variable that decreased favorable outcome in 2014 more than in 2013 and 2012; the multivariate analysis shows that this effect persists regardless of injectable drug use and additional resistance profiles. For the most part, the effect is due to increased loss to follow-up; this is a significant gap that the programme must urgently address.

In common with any other operational research study which takes advantage of available programmatic data, there are certain limitations to take into consideration. This is a single-cohort study, with no control group.

In order to be able to define treatment outcome, treatment initiation and termination dates are needed; healthcare facilities are required to report these dates, and if this is not performed adequately, not all cases can be evaluated. This situation was more common in the provinces than in Lima-Callao. Thirty percent of patients were excluded as “not evaluated”; however, no significant differences that compromise generalizability were seen between the groups, and therefore the study is considered representative of Peru. The National Resistant Tuberculosis Registry includes no treatment information prior to diagnosis of drug-resistance. As treatment cards are not available, there is no opportunity for source data verification.

In Peru, cases treated for drug-resistance are required to have monthly cultures throughout the course of treatment [16], however this is not performed in all cases, particularly when cough has resolved or become non-productive, which limits the determination of “cured” status. There are no defined criteria for treatment outcome with this type of resistance, in particular for cure and failure; therefore WHO criteria were adapted for this study [20]. Given the high loss to follow-up the true failure rate is difficult to assess. An additional limitation of this analysis is that the information system does not record data on acquired resistance or post-treatment relapse.

The presence of H resistance is associated with unfavorable treatment outcomes. In a prospective observational study conducted in Lima, Peru, on 1039 cases between 2010 and 2011, 8% of cases had H resistance alone. These cases had a higher risk of failure (2%) and death (5%) than susceptible cases [22].

In other retrospective studies in which treatment regimens were assessed, treatment outcomes are highly variable. Menzies et al performed a meta-analysis based on studies conducted between 1965 and 2008, with very variable regimens, and highoighted the complete lack of randomized controlled clinical trials. In cohorts that used 2HREZS/1HREZ/5HRE with a continuation phase of daily, or 2 or 3 times a week administration, the failure rates were 18–44%. Other regimens which included R use only in the initial phase had a relapse and failure rate of up to 70%. Unfavorable outcomes were found to be related to use of R exclusively during the initial phase, and it is recommended that at least 4 effective drugs be used during the intensive phase and 3 during the continuation phase [23].

Gegia et al expanded the meta-analysis mentioned above with studies conducted up to March 2015; the use of first-line drugs to treat H resistance was evaluated. Cases which used the WHO recommended regimen for new patients (2HREZ/4HR) showed an 11% failure rate, a 10% relapse rate, and development of MDR-TB in 8%. Among those who received the WHO regimen recommended for previously treated patients (2HREZS/1HREZ/5HRE), failure rate was 6%, relapse rate was 5%, and 3% developed MDR-TB. Therefore, the development of better regimens is recommended [19].

There is a distinct paucity of data on the use of fluoroquinolones for treatment of Hr-TB though there are some encouraging early signals. In an evaluation of 40 patients conducted in Denmark from 2002 to 2007 with H resistance determined by BACTEC 460, the REZ regimen plus fluoroquinolone for an average of 277 days had a favorable outcome in 90% of cases [24].

Further evidence for the use of fluoroquinolones instead of isoniazid comes from three large clinical trials using a fluoroquinolone (moxifloxacin or gatifloxacin) with the aim of reducing treatment for drug-susceptible TB to 4 months [25–27]. Treatment outcomes achieved by fluoroquinolone regimens were non-inferior compared with those seen with conventional treatment (2RHZE4RH).

Lee et al presented treatment outcomes with fluoroquinolone use (Lfx and Mfx) in 75 cases of H resistance as determined by DST using proportion method. This regimen had R (94.7%), E (90.7%), Z (82.7%), and S (2.7%) with a favorable treatment outcome in 97.3% and only 1.3% failures [28].

Overall the use of fluoroquinolones improves treatment outcomes over non-fluoroquinolone containing regimens, but studies are scarce. Current evidence suggests that regimens for H resistant TB should at least include R throughout treatment, should include at least 4 effective drugs for the induction phase and should probably last over 6 months, perhaps with additional benefit from prolonging to 9 months. A forthcoming systematic review, undertaken to inform new WHO guidance on Hr-TB management, should shed further light on the minimum requirements for an effective regimen.

The present study evaluated a much greater number of cases (n = 947) than previously reported; favorable outcome was significantly affected by loss to follow-up, which was greater than in other studies, but failure was minimal (1.2%).

In case of no resistance to the drugs included in the treatment, the regimen indicated in Peru for treatment of isoniazid resistant TB has a high treatment success rate and does not require the addition of an injectable drug to the regimen. In programmatic conditions, with the use of rapid DST which includes an isoniazid susceptibility test, the regimen 2LfxRZE/7LfxRE has shown strong performance in patients who remained in the programme. The regimen was derived from a detailed prior analysis of national drug-resistance epidemiology in strains resistant to isoniazid but susceptible to rifampicin. Improved adherence and sustained access to rapid DST may serve to further improve treatment success. In places where the resistance profile is known in strains resistant to isoniazid but not to rifampicin, the use of rapid DST with a rapid isoniazid test is recommended, along with the use of the 2LfxRZE/7LfxRE regimen, with particular attention to minimize loss to follow-up during treatment.

## Supporting information

S1 Table Characteristics of evaluated and not evaluated groups, and based on injectable drug use. (DOCX)

S1 Dataset Revised base de datos anonymised year of treatment removed. (XLS)
