# Supplementary material for: Treatment outcomes for isoniazid-monoresistant tuberculosis in Peru, 2012-2014
Source: PLoS One. 2018 Dec 4;13(12):e0206658. doi: 10.1371/journal.pone.0206658 (PMC6279036; doi:10.1371/journal.pone.0206658)
Supplement: S1 Table — (DOCX) [file pone.0206658.s001.docx]

S1 Table. Characteristics of evaluated and not evaluated groups, and based on injectable drug use.

|  | Cases | Evaluated  n=947 |  | Cases Not | Evaluated  N=403 |  |  |
| --- | --- | --- | --- | --- | --- | --- | --- |
|  | LfxRZE  n=791 | Injectable  LfxRZE  n=156 | p | LfxRZE  n = 352 | Injectable  LfxRZE  n = 51 | p | p |
| Sex |  |  |  |  |  |  |  |
| F | 253  32.0% | 42  27.0% | 0.2 | 115  32.7% | 11  21.6% | 0.1 | 1.0 |
| M | 538  68.0% | 114  73.0% |  | 237  67.3% | 40  78.4% |  |  |
| Age |  |  |  |  |  |  |  |
| 0-14 | 19  2.4% | 3  1.9% | 0.5 | 6  1.7% | 2  3.9% | 0.02 | 0.03 |
| 15-34 | 506  63.9% | 97  62.2% |  | 263  74.7% | 28 *  54.9% |  |  |
| 35-54 | 169  21.4% | 41  26.3% |  | 52  14.8% | 15  29.4% |  |  |
| >55 | 97  12.3% | 15  9.6% |  | 31  8.8% | 6  11.8% |  |  |
| Year |  |  |  |  |  |  |  |
| 2012 | 241  30.5% | 75  48.1 % | < 0.05 | 63  18.0 % | 17  33.3% | < 0.05 | <0.05 |
| 2013 | 207  26.2% | 76  48.7% |  | 116  32.9% | 27  52.0% |  |  |
| 2014 | 343  43.3% | 5  3.2% |  | 173  49.1% | 7  13.7% |  |  |
| HIV |  |  |  |  |  |  |  |
| positive | 32  4.0% | 9  5.8% | 0.6 | 10  2.8% | 1  2.0% | 0.3 | 0.3 |
| negative | 677  85.6% | 129  82.7% |  | 297  84.4% | 47  92.1% |  |  |
| Not evaluated | 82  10.4% | 18  11.5% |  | 45  12.8% | 3  5.9% |  |  |
| Diabetes |  |  |  |  |  |  |  |
| Yes | 54  6.8% | 15  9.6% | 0.2 | 29  8.2% | 4  7.8% | 0.9 | 0.6 |
| No | 737  93.2% | 141  90.4% |  | 323  91.8% | 47  92.2% |  |  |
| Location |  |  |  |  |  |  |  |
| Lima-Callao | 474  60.0% | 140  89.7% | <0.05 | 188  53.4% | 45  88.2% | <0.05 | 0.02 |
| Provinces | 317  40.0% | 16  10.3% |  | 164  46.6% | 6  11.8% |  |  |
| Rapid DST |  |  |  |  |  |  |  |
| H resistance | 633  80.0% | 127  81.4% | 0.9 | 288  81.9% | 43  84.3% | 0.9 | 0.2 |
| Not available | 135  17.1% | 24  15.4% |  | 53  15.0% | 7  13.7% |  |  |
| Susceptible | 22  2.8% | 5  3.2% |  | 8  2.3% | 1  2.0% |  |  |
| MDR-TB | 1  0.1% | 0 |  | 3  0.8% | 0 |  |  |
| APP DST |  |  |  |  |  |  |  |
| H-resistant | 242  30.6% | 43  27.5% | 0.4 | 112  31.8% | 10  19.6% | 0.6 | 0.4 |
| HS-resistant | 259  32.7% | 57  36.5% |  | 99  28.1% | 15  29.4% |  |  |
| HSEto-resistant | 87  11.0% | 22  14.1% |  | 45  12.9% | 9  17.6% |  |  |
| HEto-resistant | 58  7.3% | 6  3.8% |  | 24  6.8% | 5  9.8% |  |  |
| Other | 19  2.4% | 5  3.2% |  | 7  2.0% | 1  2.0% |  |  |
| Not available | 126  16.0% | 23  14.7% |  | 65  18.4% | 11  21.6% |  |  |
